# Differential analysis of transient increases of serum cTnI in response to handling in rats

**DOI:** 10.1002/prp2.11

**Published:** 2013-12-05

**Authors:** Igor Mikaelian, Michael E Dunn, Diane R Mould, Gerard Hirkaler, Wanping Geng, Denise Coluccio, Rosemary Nicklaus, Thomas Singer, Micaela Reddy

**Affiliations:** 1 Hoffmann-La RocheNutley, New Jersey, 07110; 2 Projections Research Inc.Phoenixville, Pennsylvania, 19460; 3 Hoffmann-La RocheBasel, Switzerland, 4070

**Keywords:** Catecholamines, computer simulation, drug development, drug safety assessment, heart, kinetics, pharmacology, population variability, stress, ultrasensitive assay

## Abstract

Serum cardiac troponins are the key biomarkers of myocardial necrosis in humans and in preclinical species. The use of ultrasensitive assays for serum cardiac troponin I (cTnI) as a biomarker in safety studies is hampered by interindividual differences. In this study, we investigated the effect of handling procedures on serum cTnI and explored modeling and simulation approaches to mitigate the impact of these interindividual differences. Femoral-catheterized male Crl:WI(Han) rats (*n* = 16/group) were left undisturbed in their cages with no handling; subjected to 5 min of isoflurane/O_2_ anesthesia (A); or placed into a rodent restrainer followed by simulated tail vein injection (RR). Serum cTnI concentrations were assessed over a 24-h period using an ultrasensitive assay, and the study was repeated for confirmation. The mean serum cTnI concentration pre-procedure was 4.2 pg/mL, and remained stable throughout the duration of the study in the rats submitted to the A procedure. Serum cTnI concentrations increased transiently after the RR procedure with a median time to maximum concentration (*T*_max_), of 1 and 2 h and a mean maximum value concentration (*C*_max_), of 53.0 and 7.2 pg/mL in the initial and repeat studies, respectively. A population pharmacodynamic model identified interindividual, procedure- and study-specific effects on serum cTnI concentrations in rats. It is concluded that a modeling and simulation approach more appropriately describes and statistically analyzes the data obtained with this ultrasensitive assays.

## Introduction

Serum cardiac troponin I (cTnI) and cardiac troponin T (cTnT) concentrations are the cardinal biomarkers of myocardial necrosis both in the clinics and in preclinical studies (Mair et al. 1996; Thygesen et al. 2007; O'Brien 2008; Schultze et al. 2009). Until recently, baseline serum concentrations in healthy humans and animals were below the lower limits of detection (LLoD) and quantification (LLoQ) of available assays (Todd et al. 2007). With the advent of ultrasensitive cTnI and cTnT assays with LLoD and LLoQ in the sub-picogram/mL range, serum cTn concentration reference ranges have been identified in multiple species (Todd et al. 2007; Schultze et al. 2008, 2009; Mikaelian et al. 2009; Herman et al. 2011). The mean serum cTnI concentration is below 10 pg/mL in healthy naïve Wistar, WKY and Sprague-Dawley rats, and ∼18 pg/mL in healthy naïve Fisher rats (Herman et al. 2011).

The United States Food and Drug Administration recently outlined the context of use of serum cTn as a bio-marker of drug-induced cardiac injury in the major preclinical species (Woodcock 2012). This guidance is supported by key datasets published over the past 5 years that include the comparison and validation of the commercially available assays (O'Brien et al. 2006; Apple et al. 2008; O'Brien 2008; Schultze et al. 2008; Berridge et al. 2009; Newby et al. 2011), reference ranges (Todd et al. 2007; Apple et al. 2008; Schultze et al. 2008, 2009; Mikaelian et al. 2009; Herman et al. 2011), the kinetics of cTnI (Dunn et al. 2011), and assessment of serum cTnI concentrations in a variety of animal models of cardiotoxicity (Wallace et al. 2003; York et al. 2007; Mikaelian et al. 2009; Clements et al. 2010; Schultze et al. 2011). A key contribution of these studies is the identification of a short half-life for serum cTnI, which is ∼1 h in the rat and 2 h in the dog. Therefore, serum cTnI concentrations need to be assessed soon after cTnI is anticipated to be released by cardiomyocytes. In the case of minor cardiac disturbances, this assessment may need to be performed within hours of drug administration. This window of time during which serum cTnI concentrations may be used to identify a cardiac effect may be hours for compounds and procedures causing acute cardiac perturbations, to weeks for compounds causing progressive cardiac damage, as is the case following the administration of anthracyclins (Della Torre et al. 1996; Koh et al. 2004). As a result, a single blood sample drawn 24 h after a minor cardiac insult may miss a transient serum cTnI concentration increase.

When measured with ultrasensitive assays, serum cTnI data identify time-dependent fluctuations in individual patients and rats (Schultze et al. 2008; Wu et al. 2009; Herman et al. 2011), which, in the case of the rat, may be related to underlying spontaneous cardiac disease, age, the handling procedures, jugular catheter implantation, previous administrations of cardiotoxic compounds, and a telemetry device chronically implanted in the left ventricle (Schultze et al. 2009; Clements et al. 2010; Herman et al. 2011). These interindividual differences challenge the use of traditional statistics for the interpretation of serum cTnI concentration data. Traditionally, serum cTn concentrations in preclinical studies were assessed using the same criteria and statistical tools as for other biomarkers. In our laboratory, this assessment in rodents consists of a comparison with the vehicle-treated rats at the time of necropsy in the context of the internal reference range. Subsequently, other statistical approaches were proposed, including the exclusion of high serum cTnI concentrations from the analysis and the application of arbitrary thresholds to identify increases of serum cTnI concentrations (Schultze et al. 2009; Herman et al. 2011). In this context, a modeling and simulation approach as proposed herein may more appropriately describe the data while providing a valuable tool for statistical analysis.

Here, we show that serum cTnI concentrations were increased for up to 6 h in most rats subjected to simulated tail vein injection, and in a few rats left undisturbed in their cages. In contrast, serum cTnI concentrations remained stable for 24 h in rats subjected to 5 min of isoflurane/O_2_ anesthesia (A). Tail vein injection and isoflurane/O_2_ anesthesia were assessed here because they are common modalities used in preclinical drug safety studies and as a follow-up to the assessment of the effect of oral gavage on baseline serum cTnI concentrations (Schultze et al. 2009). These data suggest that the routine method of handling rats for intravenous dosing may complicate the analysis of serum cTnI concentrations up to 12 h after an intravenous administration. Also, the modeling and simulation tool presented here may provide a novel method for the analysis of serum cTnI concentrations in the context of time-course studies.

## Materials and Methods

An initial study was performed on the effects of handling on cTnI concentrations, and was followed by a second study for confirmation. Several statistical and modeling methods of analysis were used to interrogate the data in light of the interindividual and interstudy variability.

### Animals

Male Wistar rats (Crl:Wi(Han)) aged 9 weeks (225–250 g) were obtained from Charles River Laboratories (Raleigh, NC). Approximately 1 week prior to each study, the rats were implanted with a femoral catheter externalized in the interscapular region. The rats were single-housed in polycarbonate solid bottom cages in a controlled environment (temperature maintained at 22 ± 2°C and humidity at 50 ± 20%) with ad libitum access to Purina Certified Rodent Diet #5002-9 (pellets) and reverse osmosis filtered water. The catheters were exteriorized from each cage using a jacket and protective spring device, accommodating serial blood collections independent of animal handling.

Both studies were identical except that all procedure groups were housed in a single animal room for the first study and that each procedure group was housed in a separate animal room for the second study. The groups were housed in separate rooms in the second study in order to limit the possible effects of vocalization during the rodent restrainer procedure on the stress levels of the rats subjected to the anesthesia and no handling (NH) procedures and to reduce the number of technicians working in each animal room. In addition, the first study included a group administered isoproterenol as a positive control.

All experiments were conducted in accordance to the guidance of the Roche Animal Care and Use Committee. The Nutley site of Hoffmann-La Roche, Inc., is accredited by the Association for Assessment and Accreditation of Laboratory Animal Care International.

### Procedures

Following a 2-day acclimation period after shipment, catheterized rats were randomly assigned to a procedure group (*n* = 16) entailing: NH, anesthesia by placement into an isoflurane/O_2_ induction chamber for 5 min (A); restraint in a rodent restrainer and simulated tail vein injection (RR), or in the first study 0.1 mg/kg isoproterenol administered subcutaneously (catalog #16504; Sigma-Aldrich, St. Louis, MO; *n* = 8) (Fig. S1). The rats in the NH group were left undisturbed in their cages for the duration of the study. The rats in the RR group were placed into a tubular translucent plexiglass rodent restrainer. To mimic blood sampling or intravenous injection, the rats’ tail was immersed in a warm water bath for 120 sec for visualization of vasculature. A 25 gauge needle was then inserted into the tail vein for ∼10 sec. Rats subjected to the RR procedure typically vocalized when placed into the rodent restrainer and at skin puncture during the simulated venipuncture. Following the procedure or subcutaneous isoproterenol administration, all rats were immediately returned to their cages and were left undisturbed for the remainder of the studies.

### Blood collection and cTnI assessment

For all groups in both studies, with the exclusion of samples taken at necropsy, blood samples were collected through the femoral catheter. Due to blood volume limitations, a sparse blood sampling strategy was used (∼0.5 mL drawn per sample). There were two blood sample collection schemes, with eight rats/scheme for all groups except the isoproterenol group, which had two rats/scheme. The first scheme was: 0 (i.e., prior to procedure initiation), 0.083, 1, 4, and 12 h. The second scheme was: 0, 0.5, 2, 6, and 24 h. For the second study, a 24-h sample was obtained from the rats in the first scheme in the animal room, prior to transport to the necropsy room. In addition, in the second study, blood was collected from all rats from the abdominal aorta at necropsy.

Blood samples were collected into labeled serum separator tubes and allowed to clot for at least 30 min at room temperature. Following cold centrifugation, serum samples were transferred to vials and stored at −70°C until analysis.

Serum samples (50 μL each) were run in duplicate, and serum cTnI concentrations were quantified according to the manufacturer's recommendations using the Singulex Erenna® Ultrasensitive Immunoassay system (Singulex, Alameda, CA). Final serum cTnI concentrations as reported herein represent the mean concentration of the duplicate serum samples. Serum cTnI concentrations below LLoQ (0.8 pg/mL) were reported as “LLoQ.” These concentrations were replaced by a concentration of 0.4 pg/mL for the statistical analysis. There were no serum cTnI concentrations below LLoD.

### Histopathology

Necropsies were performed immediately after the 24-h blood collection. At necropsy, the rats were anesthetized with isoflurane/O_2_ anesthesia and exsanguinated by catheterization of the abdominal aorta followed by a pneumothorax. The heart was fixed in 10% neutral-buffered formalin and processed for histology. Three longitudinal sections of the heart ∼2 mm apart were stained with hematoxylin-eosin for histomorphologic evaluation.

### Analysis of cTnI data

Our internal reference range for serum cTnI concentrations in blood collected from the retro-orbital sinus in naïve, 7–9 week-old, isoflurane/O_2_ anesthetized male rats is 2.9 ± 6.4 pg/mL (mean ± SD; *n* = 359), with a range of 0.8–96.1 pg/mL, with 97.5% of the concentrations below 11.8 pg/mL and 96.1% below 10 pg/mL. Therefore, a concentration of 12 pg/mL was considered the threshold for making a determination of increased serum cTnI concentrations. A serum cTnI concentration of 4703 pg/mL in one rat at necropsy was considered the result of trauma to the heart during the exsanguination process and therefore this data point was not included in the analysis.

The data are reported as mean, median, and range for each group/time point. Serum cTnI concentration data were evaluated independently for each study (Table 1) and were also combined to estimate a mean baseline value prior to procedure initiation across groups. The serum cTnI concentrations for each animal were fitted to an analysis of variance model using GraphPad Prism (Version 5.02; GraphPad Software Inc., La Jolla, CA) and compared to serum cTnI concentrations prior to procedure initiations for the three handling procedures using the Kruskal–Wallis test, and then to the NH group using Dunnett's test. For the isoproterenol group which had a variance different from that of the other procedure groups, serum cTnI concentrations over time were compared to serum cTnI concentration at *T*_0_ using Dunnett's test.

**Table 1:** Serum concentrations of cardiac troponin I (cTnI, pg/mL) as a function of time (h) in serum samples of femoral-catheterized rats left undisturbed in their cage no handling (NH), subjected to isoflurane/O_2_ anesthesia (A), placed in a rodent restrainer and subjected to a simulated intravenous injection (RR), or administered isoproterenol (I)^3^

Group	NH		A		RR		I
				
Study	1	2	1	2	1	2	1
0^1^	2.9 (1.8) [1.0–8.9]	6.5 (1.6) [LLoQ–79.1]	4.6 (4.0) [1.5–9.8]	2.8 (2.0) [1.2–7.4]	4.2 (2.8) [1.3–23.0]	3.4 (3.0) [1.5–6.3]	6.2 (3.9) [1.0–21.8]
0.083^1^	19.9 (8.0)** [2.8–62.]	6.6 (1.7) [LLoQ–41.6]	3.7 (2.5) [1.2–9.8]	2.4 (2.4) [1.3–3.4]	17.5 (9.7) [6.2–59.4]	4.4 (2.7) [1.2–17.2]	12.7 (9.3) [6.5–22.3]
0.5^1^	5.4 (3.9) [1.2–16.1]	2.0 (1.6) [0.81–4.1]	2.8 (2.0) [1.0–6.1]	3.1 (2.5) [1.5–5.7]	32.9 (23.9)**^,^^#^ [2.5–85.8]	4.6 (4.2)^#^ [1.7–7.6]	619 (599) [295–981]
1^1^	6.2 (3.2) [1.4–28.7]	6.4 (2.9) [1.5–32.1]	3.9 (3.4) [1.7–7.0]	2.2 (2.4) [LLoQ–3.6]	74.9 (51.8)***^,^^##^ [8.7–210.0]	9.4 (7.1) [1.4–24.7]	3280 (1864)* [696–8698]
2^1^	2.9 (2.1) [1.3–7.1]	1.9 (1.6) [1.0–3.2]	3.7 (3.0) [1.2–7.2]	3.7 (3.1) [1.2–7.3]	40.0 (20.7)***^,^^#^ [4.6–128.3]	4.6 (4.0)^#^ [2.4–7.5]	4569 (4596)*** [3037–6046]
4^1^	3.0 (2.5) [1.5–6.8]	4.8 (2.6) [1.1–17.5]	2.5 (2.2) [1.2–4.2]	2.5 (1.9) [LLoQ–6.6]	17.5 (4.3) [2.69–58.6]	3.6 (3.8) [1.9–5.5]	4121 (2914)** [1022–9632]
6^1^	8.9 (1.7) [1.0–60.3]	1.7 (1.5) [0.8–2.9]	3.0 (2.7) [LLoQ–6.8]	2.5 (2.1) [0.9–6.9]	8.8 (6.0) [2.3–18.1]	2.2 (2.4) [1.4–2.7]	3861 (3354)** [2303–6434]
12^1^	5.5 (2.1) [LLoQ–18.5]	1.9 (1.5) [LLoQ–5.6]	1.8 (1.5)** [LLoQ–4.3]	1.8 (1.6) [LLoQ–3.8]	3.6 (2.2) [1.4–9.8]	2.0 (1.8) [1.3–3.3]	1289 (487) [115–4067]
24^1^	3.1 (2.7) [1.1–6.2]	2.2 (2.2) [1.5–3.0]	10.8 (3.9) [2.0–46.4]	3.1 (2.5) [LLoQ–7.5]	3.4 (3.1) [1.9–5.5]	4.0 (3.8) [1.1–7.9]	600 (512) [212–1163]
24^2^	–	2.7 (2.3) [1.0–5.2]	–	3.5 (3.1) [1.3–7.8]	–	3.5 (3.0) [1.2–6.9]	–

1 Blood collected via the femoral catheter in the animal room.

2 Blood collected via the abdominal aorta in the necropsy room.

3 The data from the two studies are reported as Mean (Median) [Min–Max]; at time = 0 h *n* = 14–16 for NH, A, and RR and *n* = 7 for I in each study, and at other times up to 12 h *n* = 6–8 for NH, A, and RR and *n* = 3–4 for I in each study, and at time = 24 h *n* = 8–16 for NH, A, and RR in each study; serum concentrations of cTnI were compared to baseline (*P < 0.05, **P < 0.01, ***P < 0.001) and to concurrent NH controls (#*P* < 0.05; ##*P* < 0.01).

Standard non-compartmental evaluation of pharmacokinetic (PK) parameters from serum concentrations was performed using Phoenix® WinNonlin® version 6.2.0 (Pharsight, a Certara™ Company, St. Louis, MO). The primary cTnI parameters reported are maximum serum concentration (*C*_max_), the area-under-the-concentration-time curve (AUC), from zero to 24 h (AUC_24h_), and time to maximum concentration (*T*_max_). The average concentration over the 24-h period, calculated as AUC_24h_/24 h, was also reported. For study 2, parameter values based on individual animals were calculated and summarized (mean, median, and range, except for *T*_max_, for which only median and range were reported). But for study 1, the AUC_24 h_ had to be calculated using composite data because there were no cTnI concentrations after 12 h in half the animals.

### Pharmacodynamic model analysis

Data for the NH, A, and RR procedures from both studies were combined into one database for evaluation. The final database contained 504 serum cTnI concentration measurements from 96 rats undergoing the NH (*n* = 32), RR (*n* = 32) and A procedures (*n* = 32). The effect of handling on serum cTnI concentrations was described using a pulse input function as a “kinetics of action-pharmacodynamic” (K-PD) function (Jacqmin et al. 2007). Thus, the effect was treated using an exponential function:

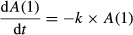
(1)where A(1) is the handling effect and k is the rate constant of decay of the handling effect which was fixed to 3 to ensure the initial effect would wear off rapidly. The effect of handling was described using an *E*_max_ function:

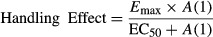
(2)where *E*_max_ is the maximum effect from handling and EC_50_ is 50% of the empirical forcing function required to achieve half maximal effect. *E*_max_ was estimated separately for each type of handling but EC_50_ values did not need to be separated.

The time course of serum cTnI concentration following handling was described using an indirect effect model (Dayneka et al. 1993) where the effect of handling stimulated the formation of cTnI using the following function:

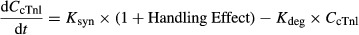
(3)where *K*_syn_ is the zero order synthesis rate constant for serum cTnI concentration and *K*_deg_ is the first order rate constant of loss. At steady state *C*_cTnI_ is equal to the ratio of *K*_syn_ to *K*_deg_. Between subject variability was described on the baseline, *K*_deg_ and *E*_max_ using an exponential function.

The data were fit using a log transform both sides approach with an additive residual error of the following function:

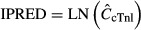
(4)

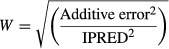
(5)

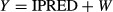
(6)where IPRED is the ln-transformed value of the individual predicted cTnI concentration, *W* is the standard deviation of the residuals and *Y* is the estimated serum cTnI concentration. The residual error was estimated separately for each of the two studies in the database.

Nonmem 7.2 version 1 (Boeckmann et al. 2006) compiled under Intel Fortran 10.1.1 (Santa Clara, CA) on a Dell quad pro quad server system running Windows Server 2007 x 64 was used for this evaluation and R 2.12.1 was used for graphical evaluations. The first order conditional estimation method (FOCE) was used.

## Results

### Observed cTnI concentration data

**Figure 1:**
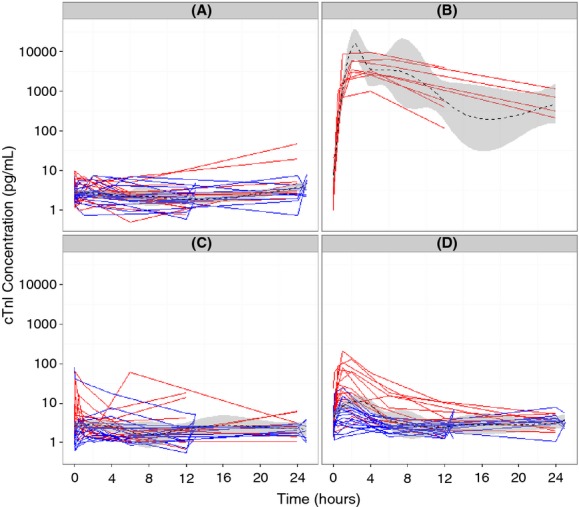
Plot of serum cardiac troponin I (cTnI) values over time by procedure for study 1 (red) and study 2 (blue) for the anesthesia (A), isoproterenol (B), no handling (C), and rodent restrainer groups (D). The solid lines represent values for individual animals and the dashed lines are lowess smooths of the observed data with the gray areas designating 95% confidence intervals. Serum cTnI concentrations increases were pronounced in magnitude and duration in rats administered isoproterenol (B). They were minimal in magnitude with a return to pre-procedure concentrations at 6 h for the rodent restrainer group (D). They were minimal in magnitude and short-lived for the no handling group (C) and absence for the anesthesia group (A).

Serum cTnI concentrations were high in one rat from the NH group at *T*_0_ (79.1 pg/mL), and increased at the 6 and/or 24-h time points in individual rats subjected to the NH and A procedures. The rat with serum cTnI concentration at 79.1 pg/mg at *T*_0_ had high serum cTnI concentrations at the 5 min (41.6 pg/mL), 1 h (32.1 pg/mL), and 4 h (17.5 pg/mL) time points, and serum cTnI concentrations of ∼5 pg/mL at the 12 h and 24-h time points. These instances of high serum cTnI concentrations were considered unrelated to the procedure because they occurred either before the procedure or late in the study. However, these data points were included in the summary table (Table 1). Data for individual rats are shown in Figure 1, which also depicts the central tendency of the data using a lowess smooth of the observed data with 95% confidence intervals.

Mean serum cTnI concentration at *T*_0_ was 4.2 ± 8.2 pg/mL (mean ± SD), with a range of LLoQ-79 pg/mL and a median of 2.4 pg/mL. The major finding of this study consisted of increases of serum cTnI concentrations in rats subjected to the RR procedure (Fig. 1; Table 1; Table S1). These increases were of greater magnitude and affected more rats in the first study than in the second study. In the first study, 12 of 16 rats had increased serum cTnI concentrations (12.4–210 pg/mL) for at least one time point, with a clear temporal pattern characterized by the onset of increased serum cTnI concentrations at 30 min and a return below 12 pg/mL at 6 h in most rats, and a return to below the threshold in all rats at 12 h. In the second study, serum cTnI increases above threshold occurred in 3/16 rats and had a smaller magnitude (14.9–24.7 pg/mL).

Serum cTnI concentrations above threshold were of lesser magnitude (13.9–62.1 pg/mL) for at least one time point in 6 of 16 rats subjected to the NH procedure in the first study (Fig. 1). These increases occurred between the 5 min and 1-h time points, and returned to below threshold at 2 h. In contrast, serum cTnI concentration increases were not identified in the rats subjected to the NH procedure in the second study. There were no serum cTnI increases in the rats subjected to the A procedure. Serum cTnI concentrations in rats administered isoproterenol followed the pattern described in the literature and peaked at 2–6 h after dosing (Table 1) (Mikaelian et al. 2008; Clements et al. 2010).

Histomorphologic changes were present only in the rats administered isoproterenol and consisted of myocardial necrosis similar in distribution and severity to the findings described in the literature (Mikaelian et al. 2008; Clements et al. 2010). Serum cTnI concentration changes in all other groups occurred in the absence of a histomorphologic correlate. In particular, there was no myocardial necrosis, and the incidence of minimal individual cardiomyocyte necrosis was within historical range for rats of that age and strain at our laboratory, and did not correlate with increased serum cTnI concentrations.

### Noncompartmental analysis

The mean *C*_max_, AUC_24h_, and time-averaged cTnI serum concentration, *C*_ave_, were all highest in the RR group of study 1 (Table 2). For the A group in each study, the mean concentration at time zero was very similar to the *C*_ave_ (i.e., 4.6 vs. 4.5 pg/mL for study 1, and 2.8 vs. 2.8 pg/mL for study 2, respectively), emphasizing the stable baseline for this group. Interestingly, except for the rats administered isoproterenol, the *T*_max_ for all groups tended to be close to the beginning of the study, ranging from a median value of 0.29 h for the study 1 rats of the NH group to 3 h for the study 2 rats in the A group (Table 2). Despite the low median *T*_max_ values, some rats did have a *T*_max_ at 24 h.

**Table 2:** Noncompartmental analysis of cardiac troponin I (cTnI) concentrations in serum samples of femoral-catheterized rats administered isoproterenol (I), left undisturbed in their cage, subjected to isoflurane/O_2_ anesthesia (A), or placed in a rodent restrainer and subjected to a simulated intravenous injection.^1^

Group	NH		A		RR		I
				
Study	1	2	1	2	1	2	1
*C*_max_, pg/mL	16.1 (6.2) [2.3–62.1]	8.2 (3.2) [1.4–79.1]	9.2 (5.2) [2.0–46.4]	4.6 (4.8) [1.3–7.4]	53.1 (25.4) [4.3–210.0]	7.7 (6.0) [2.8–24.7]	4438 (3399) [1022–9632]
AUC_24 h_, pg h/mL	126.1^2^	63.6 (46.9) [27.5–270.0]	108.8^2^	66.4 (68.3) [20.2–123.6]	259.0^2^	78.9 (71.9) [43.3–147.5]	48,483^2^
*T*_max_, h	(0.29) [0–24]	(2) [0–24]	(1) [0–24]	(3) [0–24]	(1) [0.083–6]	(1.5) [0–24]	(4) [2–6]
*C*_ave_, pg/mL	5.3^2^	2.6 (2.0) [1.1–11.2]	4.5^2^	2.8 (2.8) [0.8–5.2]	10.8^2^	3.3 (3.0) [1.8–6.1]	2020^2^

1 Serum cTnI concentrations are calculated for individual animals and reported as (Median) [Min–Max] for *T*_max_ and Mean (Median) [Min–Max] for other parameters unless otherwise noted.

2 Calculated using composite data for reasons in the discussion section.

### K-PD model analysis

The parameter estimates for the model had good precision with the exception of the term describing between rat variability on the degradation rate constant (Table 3). The precision for this parameter was poor, but this appears to be due to the high shrinkage associated with the parameter suggesting that there was not much information available to estimate the between rat variability. However, the model performance was substantially worsened when this variability term was removed, suggesting that there is variability in the degradation of cTnI that needs to be accounted for in the model. Thus, the estimate should be considered preliminary and requires confirmation.

**Table 3:** Parameter estimates for the model of serum cTnI concentrations

Parameter (units)	Typical value	SD (%)	Parameter (units)	Between animal variability	SD (%)
Baseline (pg/mL)	2.51	3.6	PPV_baseline_,%^1^	34.35	25.8
K_deg_ (h^−1^)	0.616	7.7	PPVK_deg_,%^1^	7.5	750
E_max_ for NH group	0.511	26.8	PPVE_max_,%^1^	193	18
E_max_ for A group	0.0927	21.8			
E_max_ for RR group	7.77	32.3			
EC_50_, pg/mL	0.089	36.6			
Study 1 Additive error (pg/mL)	0.617	8.7			
Study 2 Additive error (pg/mL)	0.452	6.9			

1 PPV_baseline_, population parameter variability around the baseline; PPVK_deg_, population parameter variability around *K*_deg_; PPVE_max_, population parameter variability around *E*_max_.

**Figure 2:**
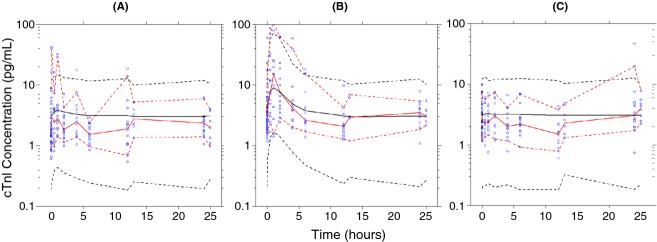
The visual predictive check of the model for the (A) no handling, (B) rodent restrainer, and (C) anesthesia groups. The open symbols are observed data, the solid red line is the median observed value, the dashed red lines describe the upper and lower 95 percentiles of the observed data, the solid black line is the median of the simulated data and the dashed black lines represent the upper and lower 95 prediction intervals.

The basic goodness of fit plot showed that the typical serum cTnI increase generally was less than 10 pg/mL (Fig. S2A), which is less than the proposed 12 pg/mL threshold value. There was good agreement between the observed and individual predicted values (Figure S2B), with significant interindividual differences in the amplitude of the response particularly for the NH and RR groups. Also, there was no apparent bias in the model, based on the diagnostic plots showing the relatively even distribution of conditional weighted residuals around zero when examined as functions of individual predicted concentration and time (Fig. 2C–D). The visual predictive check of the model stratified by procedure indicated that the model reproduced the serum cTnI response of each procedure (Fig. 2), although the lower 95% prediction interval underestimated the lower 95 percentile of the observed data across groups which is likely attributable to the need to use an additive residual error model together with the relatively small numbers of animals in the database.

This K-PD model was used to identify differences between groups. The *E*_max_ value, a measure of the handling effect, was highest for the RR group (7.77), a middle value for the NH group (0.511), and lowest for the anesthesia group (0.0927), which suggests that anesthesia may reduce the effect of handling (Table 3). Additionally, the variability was higher for study 1 than study 2.

The utility of the handling model structure is that it works for a range of responses. Even in the RR group, some animals did not exhibit the typical increase in serum cTnI concentrations, instead exhibiting a more stable baseline (Fig. 1). But the K-PD model proposed was able to describe a variety of responses by allowing interindividual variability around the *E*_max_ term (i.e., PPVE_max_ [population parameter variability] Table 2). This variability was 193%, indicating extensive variability in the cTnI response within each handling group which is reflective of the range of responses seen in Figure 1.

## Discussion

The major finding of this study was that the RR procedure was associated with serum cTnI concentration increases that would compromise the interpretation of this parameter for intravenously administered compounds between 30 min and 6 h post procedure. Additional important results of this study include: (1) the absence of serum cTnI concentration increases in rats subjected to 5 min of isoflurane/O_2_ anesthesia; (2) the occurrence of fluctuations in serum cTnI concentrations in individual rats that occurred independently from the procedure; (3) differences in the incidence and magnitude of serum cTnI concentration increases between the two studies for the RR and NH procedures and (4) the identification of a K-PD model that may help characterize subtle serum cTnI concentration increases in spite of the inherent interindividual variability and short half-life of this analyte. It is important to highlight that most of the changes in serum cTnI concentrations which were captured with this ultrasensitive assay would not have been identified with conventional serum cTnI assays which typically have LLoQ in the 30–50 pg/mL range.

### Effects of handling on the observed cTnI concentration

The increase in serum cTnI concentrations in rats subjected to the RR procedures may have been elicited by an increase in heart rate, as would be anticipated to occur as a result of this procedure. However, the difference in the incidence and the magnitude of serum cTnI concentration increases between the two studies for the RR and NH procedures indicates that additional factors may have contributed to the variability. These factors, which may include differences in baseline serum cTnI concentrations and background incidence of cardiac findings between shipment groups (I. Mikaelian, unpublished data), were ruled out in this study.

The increases in serum cTnI concentrations in rats subjected to the RR procedure in both studies and to the NH procedure in the first study occurred in the absence of a histomorphologic correlate, which may indicate that the evaluation of three sections of the heart, which is more than in routine preclinical studies, did not adequately capture the type of myocardial damage associated with these procedures. This view is supported by the observation that serum cTnI concentrations may increase in individual rats administered cardiotoxic compounds in the absence of histomorphologic correlates (Schultze et al. 2008, 2009; Mikaelian et al. 2009). An alternative hypothesis to account for serum cTnI increases in the absence of histomorphologic findings is to attribute these increases to a release from the cytoplasmic pool of cTnI. The cytoplasmic pool, contrary to the myofibril-bound pool of cTnI, may be released through blebbing prior to cardiomyocyte necrosis (Hickman et al. 2010). Ultrastructural evaluation of the heart may allow for the identification of this process. However, ultrastructural evaluation was not performed in this study because the heart was used in its entirety for histomorphologic evaluation. Furthermore, it was not known if the effects would have been diffuse or localized to specific areas of the heart, thereby limiting the feasibility of an ultrastructural assessment. The methods described here are resource-intensive and may add value for cardiac endpoints other than myocardial necrosis such as cardiac hypertrophy (Mikaelian et al. 2011) and valvular damage. However, these methods are not justified in the context of toxic myocardial necrosis, which is associated with serum cTnI increases several hundred fold in magnitude and duration greater than that caused by the RR procedure.

This study identified isoflurane/O_2_ anesthesia as a suitable method to handle rats in the context of the assessment of serum cTnI concentrations. An important rationale for repeating the first study was to confirm the absence of serum cTnI concentration increases in the A group in contrast to individual rats from the NH group. A possible hypothesis to account for this observation is that isoflurane, because of its cardioprotective properties (Shrivastav et al. 1976; Kirstetter et al. 1997; Leucker et al. 2011), may have spared the rats in the A group from environmental factors that affected the NH group. However, serum cTnI concentrations did not increase in the NH group during the second study, and therefore the role of anesthesia in preventing this increase during the first study remains speculative.

The occurrence of individual rats with high serum cTnI concentrations independent from the procedure may confound the interpretation of preclinical cardiotoxicity studies which typically are less powered than the studies presented here. These individual rats with high serum cTnI concentrations cannot be identified prior to animal placement on study because of the time and blood volume constraints of rodent preclinical studies, and because of the short half-life of serum cTnI (Dunn et al. 2011). Of note, the occurrence of animals and shipment groups with high baseline serum cTnI concentrations are rare in the two other major toxicity species, the beagle dog and the cynomolgus monkey, which simplifies the analysis of serum cTnI in these species (Todd et al. 2007; Dunn et al. 2012).

### Utility of the K-PD model

Serum cTnI concentration data in rats have historically been analyzed using several methods including: (1) comparing concentrations between treated and control groups (Mikaelian et al. 2009; Clements et al. 2010); (2) comparing concentration data to a statistical threshold calculated using historical data; (3) removing outlier datapoints from the analysis (Herman et al. 2011); or (4) log transforming the data (Schultze et al. 2009, 2011). In larger species, the comparator is the pre-dose concentration (i.e., the baseline) (Dunn et al. 2012). These approaches may overlook minor serum cTnI concentration increases. A potential limitation of some of these approaches is that information may be lost, for example, by looking only at values higher than the threshold, and an indicator of minor cardiac effects might be overlooked. We present here an alternative approach of a K-PD model that takes into consideration the baselines of individual rats and the kinetics of serum cTnI to better characterize transient fluctuations in serum cTnI concentrations.

In this study, serum cTnI concentration measurements were taken at times ranging from *T*_0_ to 24 h after the procedure. This time-course data allowed the generation of a baseline model describing changes in serum cTnI concentrations over time arising from different handling procedures, an approach that can be used to enable a PK/PD assessment in future studies of cardiac effects of test compounds. Although the baseline measurement often refers to a measurement at *T*_0_ or at some time before the study begins, the transient increase in mean serum cTnI concentrations in the RR group suggests that in this case a different definition of baseline might be more appropriate. In the case of PK/PD analysis, where the effect of a drug needs to be differentiated from a baseline value, it is important to consider the stationarity of the measured PD parameter, that is, whether statistical parameters such as mean and standard deviation change with time (Mager et al. 2003). Characterizing the complete baseline when it changes with time is important, and simply subtracting the baseline is not sufficient because of variability, for example, individual fluctuations; instead, the time-varying baseline should be built into the model using the approach illustrated here (Gabrielsson et al. 2010). This approach allows separation of the effects of the system (e.g., from the handling effect) and the drug.

PK/PD modeling is often used to identify a drug effect on a PD parameter when the baseline changes with time. In one classic population PK/PD example, the effects of disease progression and the placebo effect had to be included in the baseline model to understand the dose-response between tacrine and Alzheimer disease assessment scale in Alzheimer patients (Holford and Peace 1992). In a study of the effects of moxonidine in patients with congestive heart failure, the effect of disease progression on the biomarker noradrenaline and the effects of circadian rhythm on standing systolic blood pressure had to be incorporated in the baseline models (Brynne et al. 2001). Another study aimed at developing baseline models for body temperature, heart rate, and blood pressure in the rat that properly describe circadian effects (i.e., a turnover model of biorhythms) for analysis of in vivo pharmacology data; interestingly, a handling effect on body temperature had to be included in the model (Sallstrom et al. 2005). Here, we propose an approach for modeling the serum cTnI concentration baseline as a function of time after handling procedures that should allow the effects of agents being tested for cardiac effects to be more appropriately assessed because the impact of handling can be accounted for using this PK/PD model and can be separated from the effects of the test articles. We therefore believe that this model will prove useful for developing PK/PD models for potential cardiotoxicants that have a subtle effect on the serum cTnI concentration.

Such an approach is not needed for compounds that cause extensive cardiac necrosis and have large effects on serum cTnI concentration, such as isoproterenol in this study. For these compounds, the baseline variability is not important provided the serum is assessed within 24–36 h after the cardiac damage since the cTnI increase is of a large enough magnitude. However, for compounds with a more subtle cardiac effect, a PK/PD modeling approach using the baseline model proposed here (i.e., in the case of a handling effect on serum cTnI concentration) provides a tool to identify a less dramatic drug effect even with a time-varying baseline.

These modeling results suggest that as long as sufficient vehicle-control data are available, even a minor drug effect on serum cTnI concentrations could potentially be identified by an ultrasensitive assay. However, the biologic significance of small increases in serum cTnI concentrations remains undetermined, especially for increases below the 12 pg/mL threshold. The ability to measure and identify these small increases with the ultrasensitive assays is an important step to determining their biological significance. These increases of serum cTnI concentrations may have biologic significance because marginal increases of serum cTnT concentrations in humans are associated with an increased risk of a future cardiac event (deFilippi et al. 2010), and because serum cTnT concentrations in humans are considered “elevated” when above 10 pg/mL and “extremely elevated” above 25 pg/mL (Lipshultz et al. 2004). Additional studies are needed to determine the biological significance of these time-limited and/or serum cTnI increases within and a few fold above the reference range in preclinical species.

## Abbreviations

A: isoflurane/O_2_ anesthesia
AUC: area under the concentration-time curve
AUC_24h_: area under the concentration-time curve from zero to 24 h
*C*_max_: maximum serum concentration
cTnI: cardiac troponin I
cTnT: cardiac troponin T
*E*_max_: maximum effect
FOCE: first-order conditional estimation method
IPRED: ln-transformed value of the individual predicted cTnI concentration
*K*_deg_: first-order rate constant of loss
K-PD: kinetics of action-pharmacodynamic
*K*_syn_: zero-order synthesis rate constant
LLoD: lower limits of detection
LLoQ: lower limits of quantification
NH: no handling
PD: pharmacodynamic
PK: pharmacokinetics
PPV_baseline_: population parameter variability around the baseline
PPVE_max_: population parameter variability around E_max_
PPVK_deg_: population parameter variability around *K*_deg_
RR: rodent restrainer followed by simulated tail vein injection
*T*_max_: time to maximum concentration
*W*: standard deviation of the residuals
*Y*: estimated serum cTnI concentration

## Supporting Information

**Figure S1.** Following a 2-day acclimation period after shipment, catheterized rats were randomly assigned to a procedure group entailing: no handling (**no colored box**), isoflurane anesthesia (**fushia color box**); restraint in a rodent restrainer and simulated tail vein injection (**blue color box**), or in the first study 0.1 mg/kg isoproterenol administered subcutaneously (**red color box**). A sparse blood sampling strategy was used (**red bar**). The last blood sample preceding necropsy was performed in the animal room (**green color box**), prior to transportation to the necropsy room and necropsy (**black color box**).

**Figure S2.** Diagnostic plots for the kinetics of action-pharmacodynamic (K-PD) model, including the observed versus predicted cardiac troponin I (cTnI) concentration using the population parameters (A), the observed versus predicted cTnI concentration using the individual parameters (B), the weighted residuals as a function of individual predicted concentrations(C), and the weighted residuals as a function of time (D).

**Table S1.** Individual serum cardiac troponin I (cTnI) concentrations in femoral-catheterized rats administered isoproterenol, left undisturbed in their cage no handling (NH), or subjected to a 5 min isoflurane/O2 anesthesia (A) or to a simulated venipuncture procedure in a rodent restrainer (RR).
