# Supplementary material for: Differential analysis of transient increases of serum cTnI in response to handling in rats
Source: Pharmacol Res Perspect. 2013 Dec 5;1(2):e00011. doi: 10.1002/prp2.11 (PMC4186429; doi:10.1002/prp2.11)
Supplement: Supplementary file 1 — Figure S1. Following a 2-day acclimation period after shipment, catheterized rats were randomly assigned to a procedure group entailing: no handling (no colored box), isoflurane anesthesia (fushia color box); restraint in a rodent restrainer and simulated tail vein injection (blue color box), or in the first study 0.1 mg/kg isoproterenol administered subcutaneously (red color box). A sparse blood sampling strategy was used (red bar). The last blood sample preceding necropsy was performed in the animal room (green color box), prior to transportation to the necropsy room and necropsy (black color box). [file prp20001-e00011-SD1.pdf]

Study design

First study

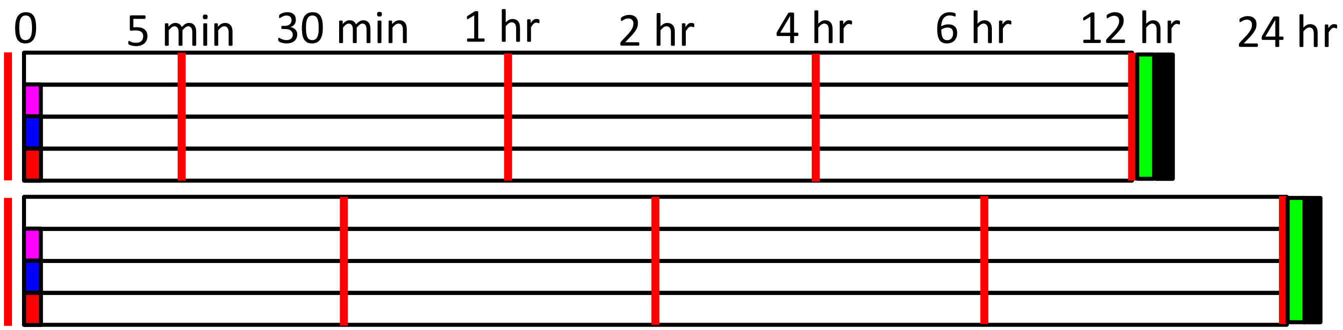

Second study

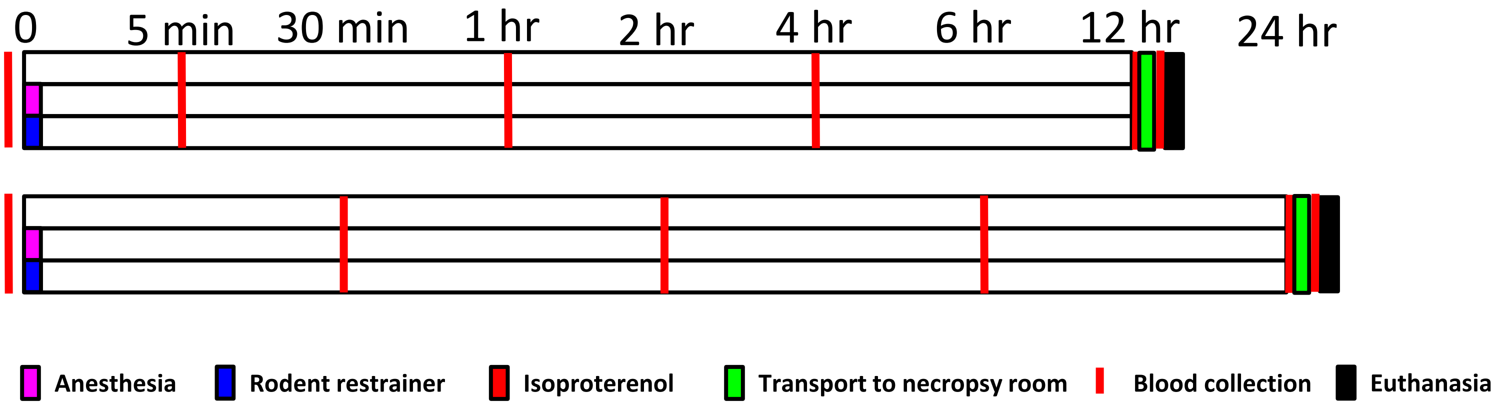

Supplemental Figure 1: Following a 2 day acclimation period after shipment, catheterized rats were randomly assigned to a procedure group entailing: no handling (no colored box), isoflurane anesthesia (■); restraint in a rodent restrainer and simulated tail vein injection (■), or in the first study 0.1 mg/kg isoproterenol administered subcutaneously (■). A sparse blood sampling strategy was used (❙). The last blood sample preceding necropsy was performed in the animal room, prior to transportation to the necropsy room and necropsy (■).
