# Supplementary figures and images for: Differential analysis of transient increases of serum cTnI in response to handling in rats
Source: Pharmacol Res Perspect. 2013 Dec 5;1(2):e00011. doi: 10.1002/prp2.11 (PMC4186429; doi:10.1002/prp2.11)

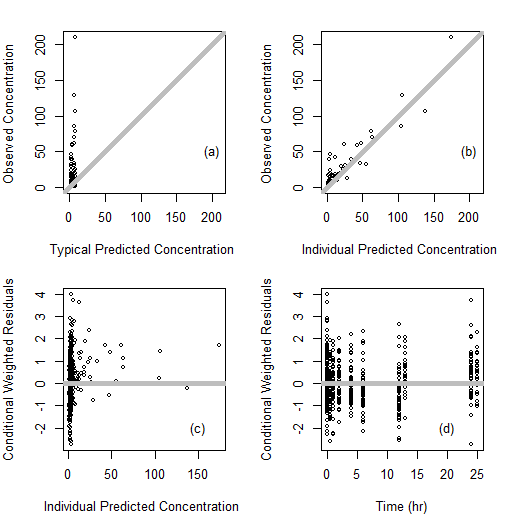

Supplement: Supplementary file 2 — Figure S2. Diagnostic plots for the kinetics of action-pharmacodynamic (K-PD) model, including the observed versus predicted cardiac troponin I (cTnI) concentration using the population parameters (A), the observed versus predicted cTnI concentration using the individual parameters (B), the weighted residuals as a function of individual predicted concentrations(C), and the weighted residuals as a function of time (D). [file prp20001-e00011-SD2.tif]
